# Ethical regulation of biomedical research in Brazil: a quality improvement initiative

**DOI:** 10.1186/s12910-024-01065-5

**Published:** 2024-06-10

**Authors:** Daniel Ribeiro Paes de Castro, Camilo Hernan Manchola Castillo, João Paulo Dias Ferreira, João Paulo Alves Oliveira, Tassila Fernandes Kirsten, Paulo Henrique Condeixa de França, Lisiane Silveira Zavalhia, Regina Kuhmmer Notti, Renata Kochhann, Sérgio Luís Amantéa

**Affiliations:** 1 Comissão Nacional de Ética em Pesquisa (CONEP), SRTV 701, Via W 5 Norte, lote D - Edifício PO 700, Asa Norte, Brasília, 70719040 DF Brazil; 2 Hospital Moinhos de Vento (HMV), R. Ramiro Barcelos, 910, Porto Alegre, 90035000 RS Brazil; 3 Av Senador Tarso Dutra 161/1303, Porto Alegre, 90690-140 RS Brazil

**Keywords:** Brazil, Consent forms, Education, Research ethics committees

## Abstract

**Background:**

Q-CEP (*Qualificação dos Comitês de Ética em Pesquisa que compõem o Sistema CEP/Conep*) is a nationwide project resulting from a partnership between the Brazilian National Research Ethics Commission (Conep), the Ministry of Health and Hospital Moinhos de Vento (HMV). It was developed to consolidate policy for ethical review of research with human beings in all members of the CEP/Conep System, Brazil’s national system of institutional review boards. The aim of this study was therefore to report on the experience and results of the Q-CEP project.

**Methods:**

An observational, retrospective study includes data from the Q-CEP, obtained from visits to all the institutional research ethics committees (RECs) in the country. The actions implemented by Q-CEP were part of a two-step process: (i) training visits to each REC; (ii) development of distance learning modules on strategic topics pertaining to research ethics evaluation. The data presented herein cover step one (training visits), defined by Q-CEP as the diagnostic stage of the project. For a country with social and economics inequalities such as Brazil, this is a particularly important stage; an accurate picture of reality is needed to inform planning of quality improvement strategies.

**Results:**

In 2019–2021, Q-CEP visited 832 RECs and trained 11,197 people. This sample covered almost all active RECs in the country; only 4 (0.5%) were not evaluated. Of the 94 items evaluated, 62% did not reach the target of at least 80% compliance and around 1/4 (26%) were below 50% compliance. The diagnostic stage of the process revealed inadequacies on the part of the RECs in their ethical reviews. The analysis of informed consent forms showed compliance in only 131 RECs (15.74%). The description of pending issues made by RECs in their reports was compliant in 19.33% (*n* = 161). Administrative and operational aspects were also considered inadequate by more than half of the RECs.

**Conclusions:**

Overall, Brazilian RECs showed poor compliance in several aspects of their operation, both in ethics evaluation and in other processes, which justifies additional training. The Q-CEP project is part of a quality improvement policy promoted by the Brazilian Ministry of Health. The data obtained in the diagnostic step of the project have contributed to the qualification and consolidation of one of the world’s largest research ethics evaluation systems.

**Supplementary Information:**

The online version contains supplementary material available at 10.1186/s12910-024-01065-5.

## Background

In recent years, there has been growing concern worldwide regarding the competence and training of members of Research Ethics Committees (RECs) tasked with conducting ethical review of clinical research projects that will involve the participation of human subjects [1–3]. This is an increasingly complex task, which requires a broad knowledge base [4] not restricted to technical or methodological details, but encompassing ethical and moral aspects as well. Research participants are intrinsically vulnerable, but the potential benefits arising from human subjects research, which can generate value and health for society, cannot be overstated.

In Brazil, a country of continental dimensions with great social, cultural, and economic diversity, an even more complex scenario is at play. The technical knowledge required to act as a member of a research ethics committee is becoming increasingly both varied and specific. Examples are provided by studies involving the use of novel drugs to treat diseases, advances in the field of genetic engineering, the use of placebo control in pharmacological studies, and ensuring post-study access to investigational medications for research participants [5–7].

Some countries have established national guidelines to guide the conduct of RECs, reflecting cultural, social, ethical, political, and even legal contexts. Although these policies may not reflect the practices actually adopted by RECs at the institutional level, they serve to encourage discussion and as a record of preferred practices of a general nature. These actions, in turn, can influence the operations of RECs. The manner in which institutional RECs were developed locally seems to explain the variation in practices observed between different countries [8–10].

In Brazil, the highest ethical authority for research involving human subjects is the National Research Ethics Committee (Conep). Established in 1996, it is a central regulatory committee which coordinates a communication network of 836 institutional RECs [11–13]. Conep is tasked with defining operational criteria for local RECs and setting all standards that guide the development of research involving human subjects in Brazil.

However, the advisory and deliberative functions carried out by Conep have become complex tasks. Standardizing the operations of 836 local RECs requires local infrastructure, training, and oversight. We are convinced that maintaining an ethical review system involves more than merely producing documents. The system must be present nationwide, operate as a network for rapid information exchange, have the ability to update itself, and ensure the safety of all subjects participating in research. These assumptions place great weight on the autonomy of individual RECs, but demand a state policy capable of supporting a central deliberative body (in Brazil, Conep) in order to ensure its stewardship role [14]. 

In its first decade of operation, the CEP/Conep system was already able to identify its needs. From the outset, a close relationship between Conep and local RECs was considered a priority for proper operation of the system. At this time, the CEP/Conep system accounted for just over 400 active RECs across the country [15]. However, the increase in Brazilian scientific output brought a corresponding increase in the number of RECs affiliated with the system [12]. There are currently 836 active RECs connected to the system operating in Brazil.

Currently, the entire ethical review process of any research involving human beings in Brazil is carried out within a digital platform called *Plataforma Brasil* (https://plataformabrasil.saude.gov.br/login.jsf). The registration and management of investigators, registration of research projects, and upload of all documents related to the research project are done on the platform, which creates a specific, individualized identifier for each project. The project evaluation process itself is also fully remote, with opinions based on the evaluation meetings held at the local RECs. All opinions issued for a given project are kept on file in chronological order. The discussion process can lead to several opinions being issued until the study is considered fit or unfit for approval. Access to the platform is password-protected and all information is stored so as to ensure privacy and confidentiality.

The Q-CEP project emerged as a response to the challenges of the then-20-year-old CEP/Conep system. The challenges faced by this structure include the distance between Conep and the local RECs, social inequalities, the growing number of RECs, the lack of professional training of committee members, and the need to strengthen the CEP/Conep system. Potential benefits of overcoming these challenges include greater institutional appreciation of RECs, harmonization of administrative procedures, improvements in ethical review of research protocols, and, as an overarching goal, greater protection of research participants [11, 13].

Q-CEP is a joint initiative of Conep and the Brazilian Ministry of Health (MoH), in partnership with Hospital Moinhos de Vento (HMV), conducted within the scope of the Unified Health System Institutional Development Support Program (PROADI-SUS). PROADI-SUS is a program in which resources arising from the tax exemption granted to nonprofit hospitals are transferred to projects whose objectives are to support and strengthen the development of the Brazilian publicly funded health system [16].

Q-CEP was devised to promote education and evaluation of the processes and infrastructure of the CEP/Conep system. It was designed throughout 2018, and scheduled to begin operations in 2019 and conclude its activities by the end of 2021.

The present article aims to report the experience and results of the Q-CEP project (*Qualificação dos Comitês de Ética em Pesquisa que compõem o Sistema CEP/Conep*, or Quality Improvement of the Constituent Research Ethics Committees of the CEP/Conep System). This project seeks to consolidate one of the world’s largest research ethics evaluation systems, which interfaces significantly with public health and patient care policies aimed at improving the health of the Brazilian population.

## Materials and methods

A cross-sectional study was designed, with information obtained from the Q-CEP Project. The Q-CEP was a nationwide project, lasting three years (2019–2021), which visited, in person or remotely, the 836 active CEPs in Brazil linked to the CEP-CONEP System. This is an observational study with data sampled retrospectively and analyzed simultaneously after the end of the visitation stages and the analysis of the improvement plans linked to the Q-CEP Project.

The design and preparation of all the stages to be developed in the project took place throughout 2018. During this period, an executive committee was set up for the project, called the *Q-CEP Trustee Committee*, made up of members from Hospital Moinhos de Vento and CONEP.

It was up to the members of HMV to select ten field researchers with experience in both ethics and research. The selection was made through a public call for applications, which was widely publicized throughout the country. All applicants were interviewed individually and had to present the required formal skills in a documented form (curriculum vitae). The ten best candidates were selected.

The CONEP team worked on the development of evaluation instruments, both for the evaluation of operational processes, as well as for the quality of ethical opinions already issued. It is important to emphasize that the CEP-CONEP System has a series of normative documents (Ordinances and Circular Letters) which seek to organize concepts relating to the assessment of ethics in research carried out on human beings in Brazil. These served as a basis for the content of the instruments.

The field researchers selected had exclusive contracts and remuneration linked to the project. At the start of 2019, the selected researchers underwent a six-month training course at Conep’s premises in Brasilia (Federal District). At this stage, they had the opportunity to expand their knowledge of research ethics. They were tutored in issuing opinions, took part in case discussions and also provided answers to questions sent to CONEP. They underwent, in an extended manner, the same training process with the activity that a new CONEP employee would be obliged to carry out, before working autonomously within the institution.

In the Q-CEP Project, all 836 active RECs were to be visited. Therefore, the sample would include the entire universe of RECs in Brazil. For operational reasons, the visitation process began in the Federal District, the geographical headquarters of CONEP, the central body regulating ethics in research carried out on human beings in the country. The selection was intentional in order to facilitate the resolution of any unexpected problems. Proximity to the central regulatory structure could facilitate their resolution. Therefore, the Federal District served as a pilot zone for the start of the project’s activities. From the Federal District, we established initial logistics for cities in the North and Midwest regions, moving on to the Northeast, South and Southeast [17]. The southeast was the last region to be visited as it had the highest concentration of RECs in Brazil. Unlike an analysis of individuals, as most surveys are structured, the Q-CEP’s unit of analysis was the REC, encompassing its infrastructure, processes and products generated.

Face-to-face visits began in August 2019. These were always carried out with the joint presence of two researchers linked to the project. The visits were pre-scheduled and the evaluation material sent at least a week in advance so that it could be answered by the local REC team. At the time of the field visit (in person or remotely), the researchers were already aware of the responses. In order for the visit to take place, we had to have confirmation of the presence of at least four members of the local REC, and the presence of its coordinator and executive secretary was mandatory.

Due to the COVID-19 pandemic, on-site assessments had to be suspended in March 2020. To avoid permanent discontinuation of the project, in July 2020, on-site visits were replaced by remote visits via web conferencing. This did not change the content of the evaluation process in any way. The team received specific training to standardize its actions and restructure documents as needed. All virtual meetings were scheduled by prearrangement, following the same sequential planning as in the original version of the project.

The assessment and training visits involved processes focused on two different dimensions of information – operational (administrative issues) and analytical (ethical reviews) – of the work carried out by each REC.

The visits were guided by standardized structured instruments, aiming to identify strengths and opportunities for improvement in all two dimensions.

Assessment of operational characteristics began before the training visits. During the visit scheduling stage (i.e., prior to the on-site visit), the REC designated for evaluation was asked to forward any documents that would be important for analysis. Therefore, before starting the assessment visit itself, the Q-CEP team already had a preliminary overview of administrative and operational aspects of the REC. This information was then confirmed or supplemented during the evaluation visit (on-site or remotely) by the Q-CEP team and the REC staff. The operational aspects covered topics, selected by Conep itself, related to REC management and performance.

The evaluation instrument was structured in the form of a 55-item checklist based on the operational standards of the CEP/Conep system. All the operating standards for a REC are set out in open access documents published by CONEP in the form of Ordinances and Circular Letters.

Processes for review of actions pertaining to ethical evaluation were not included in this assessment stage; it was limited to issues related to the REC’s composition, minimum operating conditions, physical infrastructure, relationship with Conep, and any educational activities.

To assess the analytical aspects related to the ethical reviews conducted by the RECs, a 39-item structured instrument. Again, the content of the items was based on documents and standards published by the CEP/Conep system.

The ethical review process was conducted in a completely remote fashion. Five research protocols were selected randomly from the records linked to each REC in Plataforma Brasil. After review, protocols could be placed in one of three possible adequacy categories: full compliance with the rules, partial compliance with the rules, and noncompliance with the rules.

There was no control for the complexity of the evaluated scenarios. Q-CEP investigators selected the protocols on the basis of their numeric identifier, without any prior knowledge of their content.

Both instruments (operational analysis and ethical analysis) were structured in the form of a checklist, with two possibilities for the final answer, i.e. adequacy or inadequacy to the standard established as a reference. This information served as the basis for determining compliance rates (adherence) to the standards determined as adequate, both for the process analysis and the ethical evaluation. The same instruments were used for face-to-face and remote evaluations.

The Q-CEP team sent a report of all opportunities for improvement and actions plans made jointly with REC staff at the end of the evaluation visits. The aim of this report was to establish an overview of the situation, highlighting aspects in which the REC was already compliant and seeking to identify room for improvement.

Based on this information, each REC, mediated by the Q-CEP team, was encouraged to develop an action plan for improvement. Actions for improvement could be implemented in the short term (up to 1 month after the visit), medium term (up to 6 months after the visit), or long term (up to 12 months after the visit).

The results of the evaluations were compiled and transferred to the Microsoft Excel® database. The Statistical Package for the Social Sciences (SPSS) version 25 software was used to structure both the frequency tables and the description of the categorical variables. The results were presented in absolute and relative frequencies. The compliance rates for the ideal operating characteristics (minimum proportion allocated of 80%) were compared to the compliance rates obtained for the ethical evaluation scenarios considered appropriate using the two-sample Z-test in order to determine differences in compliance. The significance level adopted was 5% (*p* = 0.05). To determine statistically significant differences, the calculated Z value had to be outside the critical range of -1.96 to 1.96. For the purposes of the study, only values below the minimum proportion assigned were considered significant, i.e. negative values outside the critical range of the Z-test.

## Results

**Fig. 1:**
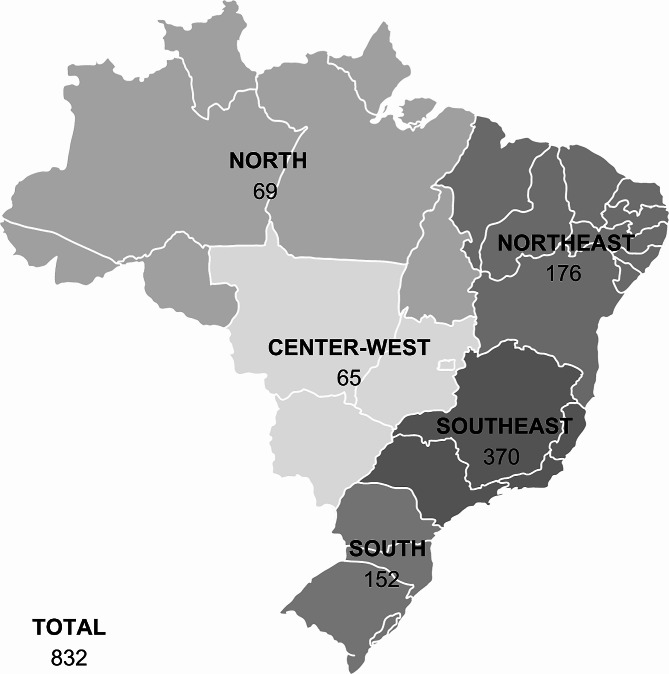
Geographic distribution of Research Ethics Committees (RECs) in Brazil

During its period of implementation, Q-CEP visited 832 RECs (259 on-site visits and 573 remote visits via web conference) in all regions and states of Brazil, including the Federal District (Fig. 1). Only four RECs (0.5%) could not be scheduled for visitation within the defined time frame. During these visits, 11.197 people were trained, including REC members and administrative staff, as well as managers of the institutions with which the RECs were affiliated. Of the total number of participants, 32% (*n* = 3582) were evaluated on site and 68% (*n* = 7615) in remote visits. A minimum of four members of the institutions under evaluation were required to be present for a scheduled visit: the REC coordinator and deputy coordinator, a member of the REC administrative staff, and a representative (director or deputy director) of the institution itself. The participation of other REC members was not only allowed but also encouraged, both in on-site and remote visits. The strategy was successful, as we assessed almost all of Brazil’s RECs (99.5%) and generated a series of real-time data valid for the entire national territory. The number of people trained per visit ranged from 4 to 43. Figure 1 shows the distribution of RECs visited by geographic region of Brazil [17].

Of the 94 items evaluated (55 operational aspects and 39 ethical review process), 62% of them did not reach the target of at least 80% compliance. In addition, around 1/4 (26%) were below 50% compliance. Thus, this diagnostic stage revealed errors and low compliance rates, both in terms of standardized operational aspects and the ethical review process.

Operational aspects of each REC were evaluated with an instrument consisting of 55 yes-or-no questions. Table 1 shows the level of compliance for three of the issues considered most sensitive, i.e., those that showed the lowest rate of compliance among the RECs in terms of administrative and operational aspects (compliance level of less than 50% compared to the response considered ideal). To compose this table, several items had very close complacencies (between 31 and 35%). We chose to include details on the review of informed consent forms, as we considered it to be a more sensitive issue than the others, given the very close complacency between the items.

**Table 1:** Compliance level for three selected issues of operational aspects (*n* = 832 RECs)

Issue	Operational standard	Level of compliance
**Submission of six-monthly activity reports to the Conep** **(Item 69 – Appendix)**	According to National Health Council Resolution No. 370/07 [18] and Operational Standard No. 001/2013 [19], the six-monthly reports of REC activities are among the main tools used by the Conep/National Health Council to understand and monitor the work of the committees. Therefore, the regular submission of reports to the Conep within the time limits established in National Health Council Operational Standard No. 001/2013 (within 2 months of every 6 months) [19] should be a well-established routine activity for the committees.	27.59% (*n* = 230)
**Updating the composition of the collegiate body of the REC** **(Item 67 – Appendix)**	Any changes in the composition of the collegiate body must be informed to the Conep and followed by the necessary alterations in Plataforma Brasil, the digital platform that remotely centralizes all processes pertaining to the CEP/Conep System. Those involved in the research (investigators, administrative members, and REC members) have individual registrations and passwords. All processes related to the conduct of research on human beings are processed in the virtual environment of the platform (registration, submissions, evaluations, and issuing of opinions). These two actions must be performed in conjunction with one another. While the changes made by the REC must be verified by Conep to ensure that they conform to the regulations of the CEP/Conep System, the REC must also update the member information on Plataforma Brasil to maintain the anonymity and confidentiality of information regarding research protocols, which should only be accessed by members of the REC.	35.65% (*n* = 297)
**Timely renewal of REC registration** **(Item 66 – Appendix)**	According to National Health Council Resolution No. 370/07 [18], RECs must renew their registration with Conep within 60 days of the expiry date of their current registration, which is valid for 3 years. The renewal must be performed within the specified period to preserve and uphold the legality of the committees and, especially, the validity of their approval of ongoing research studies.	39.32% (*n* = 328)

Table 2 shows the three questions considered most relevant regarding noncompliance with standards during ethical review, i.e., those with the lowest compliance rate among the RECs for the 39 items selected within the review process of the evaluated research protocols. As in the previous table, low levels of compliance are shown in relation to the answers considered ideal.

**Table 2:** Compliance level for three selected issues of ethical review (*n* = 832 RECs)

Issue	Importance	Level of compliance
**Information on the right to seek compensation is verified to be present in the consent forms** **(Item 16 – Appendix)**	The presence of this information is crucial to remind research participants of their human rights and legal guarantees. It is not the responsibility of the CEP/Conep System to judge or determine that anyone should provide financial compensation to a research participant. This is a matter for the legal system. However, as part of its mission to protect research participants, the CEP/Conep System must ensure that participants are informed of their right to turn to the justice system and seek compensation for any damage resulting from the study, at any point, if they so wish.	15.74% (*n* = 131)
**No specification of the CEP/Conep standards based on which ethical issues were raised** **(Item 5 – Appendix)**	Significant heterogeneity was observed in the way ethical issues were raised in research protocols in the CEP/Conep System. While variability is not a problem in itself, the fact that many reports do not reference the CEP/Conep regulations when discussing ethical issues weakens the legitimacy of the ethical review process and any requests for adjustments and clarifications. To ensure that any issues requiring further attention by researchers are adequately reported and addressed, their description must refer to the specific legislation on which the concerns are based.	19.33% (*n* = 161)
**Adequate review of informed consent forms to ensure immediate, free, and comprehensive assistance to research participants** **(Item 19 – Appendix)**	Information on these rights is of crucial importance, since individuals who agree to take part in a study must be explicitly told through the informed consent form that those responsible for the study will ensure they receive the necessary treatment for any harm or damage to their wellbeing directly or indirectly related to their participation in the study.	34.78% (*n* = 289)

Even considering the items with the lowest compliance among the RECs, we observed a significant difference between the evaluative items related to operational processes and ethical analysis. Operational processes, even considering their lower compliance rates (adherence) to the reference standard, show greater adherence to the standard considered ideal, with a difference in effect size considered significant between the proportions.

In the supplementary material included with this article, we can see the compliance rates for the 94 items evaluated, along with their statistical significance in relation to the target rate of 80% considered acceptable.

Many improvement initiatives were scheduled after the situational diagnosis had been delivered individually to each REC. Overall, the improvement plans developed by the 832 RECs proposed 8,384 actions. The plan with the most proposals for improvement contained 29 items, while that with the fewest suggestions contained only one. However, the number of suggestions should not be used as an indicator of the quality of these plans. The 8,384 proposed actions included 3,381 short-term solutions (40.32%), 3,313 medium-term initiatives, (39.51%) and 1,690 long-term improvements (20.17%). As stated in post-visit reports, RECs were asked to describe and analyze these initiatives as part of their mid-year reviews. The data provided by the RECs until November 2021 allowed us to identify the degree of implementation and success rate of these initiatives. Considering all 8,384 initiatives, 3,666 have been completed or are in progress (43.72%). Of these 3,666 initiatives, 3,124 (85.21%) were reported by RECs as having a positive effect on the overall quality of their work.

## Discussion

Although Conep had already implemented other educational and inspection activities in its 20-plus-year history, the CEP/Conep system had never developed such a comprehensive training program. An individually structured quality improvement initiative covering all research ethics committees in Brazil was thus a major challenge.

A public-private partnership, made possible by the PROADI-SUS program, Conep, the Ministry of Health, and Hospital Moinhos de Vento, made this process feasible.

Numerous educational initiatives aimed at improving the quality of REC staff training have been published internationally. However, there are no descriptions of such initiatives considering local and cultural aspects as obstacles to the implementation of improvements [18–20].

In addition to the unique nature and scope of this training effort (encompassing more than 800 RECs nationwide) and the operational planning required before the assessment and training visits, we faced other challenges. Reaching a specific diagnosis on the quality of operations of each committee was an important step before proposing any improvements. Such a result could only be obtained with the use of standardized tools, designed after well-established ethical standards, and applied by a well-trained team. This allowed us to obtain an accurate overview of key opportunities for improving the CEP/Conep system.

First begun in 2019 as on-site inspections, the Q-CEP visits were temporarily halted in March 2020 due to the COVID-19 pandemic. However, these visits soon resumed via web conference and were completed in September 2021, reaching a total of 832 local RECs.

This is a very representative sample, covering nearly all RECs operating in the country. Thus, one may infer there is a high degree of adherence to the initiative and commitment to the CEP/Conep system. All REC members are volunteers who provide a service in the public interest. The Q-CEP project trained 11.197 members.

The strategy of making the two dimensions of interest tangible in the evaluation process was also important. The levels of compliance with operational aspects, and those pertaining to ethical review, as well as the implementation of improvement actions by the RECs after the Q-CEP visits, suggest the need to: *(i)* continue monitoring the work of RECs, in order to ensure that any improvements made as a result of the Q-CEP visits are maintained; *(ii)* establish a mechanism for continuing evaluation of the quality of ethical reviews done by the CEP/Conep system as a whole, in order to allow measurement of the impact of the Q-CEP project over time; and *(iii)* develop and implement educational activities aimed specifically at improving the most common difficulties encountered by RECs.

Strengthening the CEP/Conep system entails recognizing the importance of ethical review of research involving human subjects. Ethical review cannot be perceived as a bureaucratic hindrance or just another regulatory requirement. Therefore, it is essential that those involved in such analysis have the necessary knowledge and qualifications. The first decade of operation of the CEP/Conep system was beset by difficulties, but already identified a need for quality improvement. Reports at the system’s 8-year mark of operation indicated 415 active RECs across the country, with approximately 5,000 individuals involved in the ethical review process [13].

In the year 2000, Conep evaluated approximately 1,000 research projects – around 10% of the number of projects evaluated by its constituent RECs. These figures suggest that approximately 10.000 research projects were proposed in a single year [11]. Current numbers are substantially larger. Approximately 100.000 research projects are submitted to the CEP/Conep system for appreciation each year, covering approximately 2.5 million research participants per year. The network of RECs has also expanded by approximately 60 new committees per year, with over 800 now distributed across all regions of the country [12].

In line with this growth in research output, it is extremely important to expand and strengthen the CEP/Conep system and reduce its asymmetries.

Our results revealed several opportunities for improvement, both in operational matters and in aspects pertaining to ethical review. Less compliance with attitudes considered correct was found during evaluation of ethical aspects. The three worst items among the 39 evaluated ranged from 15 to 35% compliance. We believe that this difference is intrinsic to the greater complexity of assessing ethical issues. The evaluation of processes presupposes adherence to established standards. These are related to the REC’s infrastructure, human resources, staff and user training, and the adequacy of documents and opinions. There is no subjectivity in the evaluation process, unlike ethical evaluation, which presupposes a series of other skills and competencies.

New issues and challenges can and will arise. Aspects resulting from the COVID-19 pandemic itself provide illustrative examples. Therefore, it is important for the CEP/Conep system to further mature its self-assessment capacity and establish routine procedures and tools that allow for interventions appropriate to contemporary needs.

Our results characterize the performance of the CEP-CONEP system. The regulatory system for research carried out on human beings in Brazil. It has its own characteristics that make it impossible to compare results with other ethical regulatory systems around the world. However, it has taken steps to improve the quality of the ethical evaluation process in the country. Intrinsically, the study has some methodological limitations. The most important of these is the different information acquisition processes imposed by the COVID pandemic. In any case, the project sought to minimize bias by training the assessment teams, setting up interviews with a standardized number of interviewees and mandatory executive functions, as well as using standardized data collection instruments.

## Conclusions

Q-CEP has demonstrated that educational projects can be developed as part of a supplemental integration initiative to the CEP/Conep system. In a country such as Brazil, with continental dimensions and social inequalities, it was especially encouraging to see that the implementation of this project, with its two supplemental intervention strategies (education and recognition), produced measurable changes in the quality of research ethics.

These results provided the CEP/Conep system with strategic data, which should enable creation of a list of priorities aimed at improving the quality of ethical review of human subjects research in Brazil.

For countries that use a central committee to assess and deliberate on the processes of evaluating research on human beings, this is a model that can be replicated. It is capable of generating up-to-date information that can contribute to the creation of improvement plans. In addition, it reinforces the communication network structure, which is fundamental for the qualification of research ethics evaluation.

### Electronic supplementary material

Below is the link to the electronic supplementary material.

Supplementary Material 1

## Data Availability

All data generated or analyzed during this study are included in this published article.
