# Supplementary material for: Ethical regulation of biomedical research in Brazil: a quality improvement initiative
Source: BMC Med Ethics. 2024 Jun 10;25:68. doi: 10.1186/s12910-024-01065-5 (PMC11163760; doi:10.1186/s12910-024-01065-5)
Supplement: Supplementary file 1 — Supplementary Material 1 [file 12910_2024_1065_MOESM1_ESM.docx]

**Appendix 1: COMPLIANCE LEVEL OF THE QUESTIONS CONCERNING OPERATIONAL ASPECTS AND ETHICAL REVIEW OF 832 RECs IN BRAZIL (n= 94 evaluation items)**

| **CONSOLIDATED REPORT** | **compliance**  **(%)** | **Z-test Value** |
| --- | --- | --- |
| 1. The REC filled in the "Project Presentation" field with the abstract, methodology, inclusion and exclusion criteria presented by the researcher | 50.78 | -21.07* |
| 1. The REC filled in the "Research Objective" field as presented by the researcher | 83.39 | 2.44 |
| 1. The REC filled in the "Risks and Benefits" field as presented by the researcher | 68.23 | -8.48* |
| 1. The REC recorded conclusions or pending issues in the "Conclusions or Pending Issues and List of Inadequacies" field, following the model in the Pending Issues Manual (identifies inadequate document and text, describes inadequacy, explains request). | 42.0 | -27.41* |
| 1. The REC refers specification of standards (Ethical Regulation) based on which ethical issues were raised | 19.33 | -43.74* |
| **REGISTRATION OF CONSENT** |  | |
| 1. Clear and objective language. | 68.77 | -8.10* |
| 1. It presents the justification, objectives and procedures of the research. | 64.16 | -11.42* |
| 1. Describes risks, discomforts, measures and precautions in relation to the participant. | 63.26 | -12.08* |
| 1. Describes expected benefits of participating in the study. | 74.96 | -3.99* |
| 1. Ensures the benefits resulting from the research, whether in terms of 2. social return, access to procedures, products or research agents. | 74.47 | -3.99* |
| 1. It guarantees the freedom to refuse and withdraw consent at any any stage of the research. | 89.83 | 7.11 |
| 1. It guarantees secrecy and confidentiality. | 87.57 | 5.46 |
| 1. Guarantees receipt of a signed and initialed copy of the 2. Free and Informed Consent Form / Record of Consent.. | 61.88 | -13.06* |
| 1. Informs about the right to seek compensation. (15.74) | 15.74 | -46.20* |
| 1. Provides institutional and/or home address and e-mail and contact details of those responsible for the research. | 65.93 | -10.14* |
| 1. Informs address and contact details of REC and Conep (if applicable) | 60.66 | -13.95* |
| 1. Adequate review of informed consent forms to ensure immediate, free and comprhensive assistance to research participants | 34.78 | -32.11* |
| 1. Clarifies forms of monitoring. | 35.02 | -32.45* |
| 1. Guarantees reimbursement of expenses arising from participation in the research, including the companion (if applicable). | 39.43 | -29.29* |
| 1. Explains the possibility of inclusion in a control or experimental group. | 60.71 | -13.92* |
| 1. Guarantees the supply of medicines, prophylactic diagnostic or therapeutic methods after the end of participation in the study. | 31.25 | -35.16* |
| 1. Informs about alternative therapeutic methods (if applicable) | 33.33 | -33.66* |
| 1. Guarantees assistance and follow-up for participants' pregnant partners participants and the conceptus (where applicable) | 40.91 | -28.20* |
| 1. Guarantees access to test results for the patient's doctor and/or the patient themselves whenever requested. | 39.02 | -29.54* |
| 1. Informs about the genetic counseling plan and clinical follow-up, at no cost to the participant (when applicable). | 35.71 | -31.94* |
| 1. Biorepository: Requests authorization for the collection, deposit, storage and use of human biological material in the country and/or abroad. | 36.76 | -31.19* |
| 1. Biorrepository: Informs that tests will only be carried out if 2. described in the Informed Consent Form/Register of Consent will only be carried out if authorized by the research participant. | 33.33 | -33.66* |
| **COVER PAGE** |  |  |
| 1. Checking that the cover sheet is correctly filled in (dated, signed and filled in properly). | 81.81 | 1.30 |
| **BRAZIL PLATFORM – BASIC INFORMATION / DOCUMENTS** |  | |
| 1. Verification of the pertinence of the registration of the protocol in the Conep (wrong area). | 96.72 | 12.05 |
| 1. The justification for waiving the Informed Consent Form/registration of consent was adequately accepted. | 58.21 | -15.71* |
| 1. The REC has adequately evaluated alternative ways of recording consent | 72.69 | -5.27* |
| 1. The description of the consent process is appropriate to the specific nature of the research participants. | 64.38 | -11.26* |
| 1. Biorrepository: The recipient institution's commitment in the country and/or abroad to prohibit the patenting and commercial use of human biological material. | 32.50 | -34.24* |
| 1. Biorrepository: Presented the agreement signed between the participating institutions, covering ways of operating, sharing and using the human biological material stored in the biobank or biorrepository, including the possibility of dissolving the partnership in the future and the consequent sharing and destination of the stored data and materials. | 31.40 | -35.05* |
| 1. The study schedule shows the total duration and description of the stages | 72.82 | -5.18* |
| 1. The expected start of contact (recruitment) with the participant was after the initial submission date. | 64.99 | -10.82* |
| 1. Present the relevant authorizations / declarations / consents. | 73.81 | -4.46* |
| **REC's history and information** |  | |
| 1. The REC has not been reported (and the complaint was well-founded) in the last 36 months. | 92.79 | 9.22 |
| 1. The REC has not had its registration suspended in the last 36 months. | 97.12 | 12.35 |
| 1. REC has not been deregistered in the last 36 months. | 89.90 | 7.14 |
| **Administrative management of the REC** |  | |
| 1. Does REC has an exclusive area? | 92.67 | 9.15 |
| 1. Does REC has an administrative employee? | 86.78 | 4.88 |
| 1. Does REC has computer equipment with internet access? | 97.00 | 12.26 |
| 1. Does REC has a telephone line available for contact? | 93.39 | 9.66 |
| 1. Does REC has furniture and office supplies? | 96.75 | 12.08 |
| 1. Does the institution have external signage, as established in CNS Procedural Standard No. 006/2009? | 44.71 | -25.44* |
| 1. Does the institution have internal signage, as established in CNS Procedural Standard No. 006/2009? | 75.36 | -3.35* |
| 1. Is the institution's reception able to tell you the exact location of the REC? | 72.59 | -5.36* |
| 1. Does REC has an archive to store administrative documents and projects submitted to it for a period of 5 years, as established in CNS Resolution 370/2007? | 96.88 | 12.18 |
| 1. Does REC record the meetings in an approved document, as established in CNS Resolution 370/2007? | 94.71 | 10.61 |
| 1. Are REC meetings attended by more than 50% of the members, as established in CNS Resolution 370/2007? | 94.59 | 10.52 |
| 1. The REC analyzes protocols from other institutions. | 93.39 | 9.66 |
| 1. The sponsoring institution counts the time that all REC members spend on regular Committee activities as part of their total workload. | 56.27 | -17.12* |
| 1. The sponsoring institution counts as part of the total workload the time that the REC coordinator spends on regular Committee activities. | 60.95 | -13.74* |
| 1. The REC's maintaining institution authorizes and counts as part of the members' total workload when they participate in events related to ethics in research with human beings (e.g. Conep trainings, academic meetings, research ethics congresses, among others). | 72.40 | -5.48* |
| 1. The REC's maintaining institution reimburses members when they participate in events related to ethics in research with human beings (e.g. Conep trainings, academic meetings, research ethics congresses, among others). | 74.85 | -3.71* |
| 1. The sponsoring institution has an Incentive Plan for participation in the REC (e.g. participation in the REC contributes to career progression within the institution). | 37.77 | -30.45* |
| 1. Is the REC's membership multidisciplinary? | 98.20 | 13.12 |
| 1. Does REC's membership include both genders? | 99.28 | 13.91 |
| 1. Does REC's membership include proportionality for the participation of representatives of the user population? | 76.92 | -1.52 |
| 1. Do at least 50% of REC members have experience in research? | 98.92 | 13.22 |
| 1. The REC is active (regularized). | 96.27 | 10.59 |
| 1. The last renewal of the REC's registration was completed within the deadlines established in CNS Resolution 370/2007. | 39.32 | -29.20* |
| 1. Is the composition of the REC on the Brazil Platform in line with the latest membership form sent to Conep? | 35.65 | -35.50* |
| 1. Has the REC sent Conep all the required six-monthly reports? | 42.43 | -17.49* |
| 1. Did the REC send Conep all the biannual reports within the deadline established in CNS Operational Standard 001/2013? | 27.59 | -25.33* |
| 1. Do the reports contain the qualitative information relating to the item "Meetings" in the annex to CNS Operational Standard No. 001/2013? | 79.93 | -0.03 |
| 1. The reports contain qualitative information relating to the item "Structure and functioning" in the annex to CNS Operational Standard 001/2013. | 78.49 | -0.76 |
| 1. The reports contain qualitative information relating to the item "Monitoring the development of research projects" in the annex to CNS Operational Standard 001/2013. | 72.48 | -3.61* |
| 1. The reports contain qualitative information relating to the item "Consultative and educational role of the REC" in the annex to CNS Operational Standard 001/2013. | 74.52 | -2.67* |
| 1. The reports contain quantitative information on the minimum number of projects analyzed and meetings held in accordance with CNS Resolution 370/07. | 83.53 | 1.86 |
| 1. The REC has issued 75% or more substantiated opinions within the deadlines set out in CNS Operational Standard 001/2013 in the last 12 months. | 63.94 | -7.40* |
| 1. Does REC publish the days and hours of operation of the Committee's secretariat? | 60.22 | -9.0* |
| 1. Does REC publish its calendar of meetings for the year? | 53.73 | -11.85* |
| 1. REC publishes your contact information (telephone and e-mail). | 62.98 | -7.82* |
| 1. Does REC publish the office's opening hours? | 60.46 | -8.92* |
| 1. Does REC publish the recess period with start and end dates? | 41.21 | -17.64* |
| 1. The portals are adapted for wheelchair access. | 89.60 | 5.50 |
| 1. Access routes to the REC are adapted (ramp, parking and others). | 91.20 | 6.58 |
| 1. A wheelchair is available for people with reduced mobility. | 75.81 | -2.06* |
| 1. The toilets are adapted for people with reduced mobility. | 89.37 | 5.34 |
| 1. The user representative(s) are listed as members of the REC on Brazil Platform. | 55.65 | -11.01* |
| 1. The letter of appointment for the user representative(s) is up to date. | 79.57 | -0.21 |
| 1. The user representative(s) actively contributes to the discussions and deliberations at the REC collegiate meetings. | 68.75 | -5.30 |
| 1. In the first two months of each year, the REC approves an initial and ongoing training plan for its members. | 34.25 | -21.29* |
| 1. The REC carries out educational activities for researchers. | 74.28 | -2.78* |
| 1. Provides educational material for researchers. | 64.90 | -6.98* |
| 1. The REC carries out educational activities for research participants and the community in general. | 40.75 | -17.86* |
| 1. REC members participate in events, forums, seminars and other activities related to ethics in research with human beings. | 85.22 | 2.81 |
| 1. REC liaises with other committees to fulfill its mission of to protect research participants. | 59.62 | -9.26* |
| 1. REC liaises with movements and entities that are not part of the REC/Conep system in order to fulfill its mission of protecting research participants. | 43.27 | -16.63* |

* The significance level adopted was 5% (p=0.05). To determine statistically significant differences, the calculated Z value had to be outside the critical range of -1.96 to 1.96. For the purposes of the study, only values below the minimum proportion assigned were considered significant, i.e. negative values outside the critical range of the Z-test.
